# A systematic flux analysis approach to identify metabolic vulnerabilities in human breast cancer cell lines

**DOI:** 10.1186/s40170-019-0207-x

**Published:** 2019-12-27

**Authors:** Sheree D. Martin, Sean L. McGee

**Affiliations:** Metabolic Reprogramming Laboratory, Metabolic Research Unit, School of Medicine and Centre for Molecular and Medical Research, Deakin University, Geelong, Victoria Australia

**Keywords:** Breast cancer, Metabolism, AMPK, mTORC1, Metabolic flux analysis, Metformin

## Abstract

**Background:**

Increased flux through both glycolytic and oxidative metabolic pathways is a hallmark of breast cancer cells and is critical for their growth and survival. As such, targeting this metabolic reprograming has received much attention as a potential treatment approach. However, the heterogeneity of breast cancer cell metabolism, even within classifications, suggests a necessity for an individualised approach to treatment in breast cancer patients.

**Methods:**

The metabolic phenotypes of a diverse panel of human breast cancer cell lines representing the major breast cancer classifications were assessed using real-time metabolic flux analysis. Flux linked to ATP production, pathway reserve capacities and specific macromolecule oxidation rates were quantified. Suspected metabolic vulnerabilities were targeted with specific pathway inhibitors, and relative cell viability was assessed using the crystal violet assay. Measures of AMPK and mTORC1 activity were analysed through immunoblotting.

**Results:**

Breast cancer cells displayed heterogeneous energy requirements and utilisation of non-oxidative and oxidative energy-producing pathways. Quantification of basal glycolytic and oxidative reserve capacities identified cell lines that were highly dependent on individual pathways, while assessment of substrate oxidation relative to total oxidative capacity revealed cell lines that were highly dependent on individual macromolecules. Based on these findings, mild mitochondrial inhibition in ESH-172 cells, including with the anti-diabetic drug metformin, and mild glycolytic inhibition in Hs578T cells reduced relative viability, which did not occur in non-transformed MCF10a cells. The effects on viability were associated with AMPK activation and inhibition of mTORC1 signalling. Hs578T were also found to be highly dependent on glutamine oxidation and inhibition of this process also impacted viability.

**Conclusions:**

Together, these data highlight that systematic flux analysis in breast cancer cells can identify targetable metabolic vulnerabilities, despite heterogeneity in metabolic profiles between individual cancer cell lines.

## Background

Cancer cells reprogram their metabolism to drive high rates of proliferation and ensure their survival under conditions of fluctuating nutrient availability [1]. Early characterisation of these alterations in metabolism suggested that cancer cells exclusively increased glycolytic flux to maintain high rates of ATP production [2]. It is now recognised that although glycolysis is elevated in most cancer cells, flux through oxidative metabolic pathways is often also increased [3]. Enhanced flux through both these major metabolic pathways not only maintains cellular energy balance, which is critical for maximal activity of growth signalling pathways such as mammalian target of rapamycin complex 1 (mTORC1), but also provides metabolite intermediates for the synthesis of nucleotides and lipids, as well as balancing the cellular redox state [1]. However, the metabolic phenotype of different cancer types is highly heterogeneous [4].

As cancer cells are highly dependent on metabolic reprogramming for their proliferation and survival, targeting tumours with therapies that inhibit specific metabolic pathways has been touted as a new treatment approach [5]. Indeed, a number of early phase clinical trials have utilised metabolic inhibitors as both standalone and combination therapies with existing treatments [5]. Given the heterogeneity in cancer cell metabolism, it is necessary to discover persistent metabolic vulnerabilities that can be targeted in specific cancer types [5]. A common approach has coupled stable isotope tracers to metabolomics to quantify substrate flux through various metabolic pathways [6]. Importantly, this approach can be used in vivo in both patients and pre-clinical models and it has successfully identified metabolic vulnerabilities in renal clear cell [7], lung [8], pancreatic [9] and glioblastoma tumours [10], to name a few. However, specific stable isotopes are required to interrogate particular metabolic pathways, which require some prior knowledge of the type of metabolic vulnerability being investigated [11]. It is also recognised that metabolism within an individual tumour can be spatially heterogeneous due to factors such as nutrient and oxygen penetrance [12, 13], and therefore, the site of sampling can have a profound impact on conclusions generated using this approach. Another method used to identify metabolic vulnerabilities is real-time flux analysis in isolated and cultured cancer cells [6]. Although this approach cannot account for in vivo conditions that influence metabolism, ex vivo analyses are likely to identify persistent metabolic reprogramming events that are independent of the metabolic environment yet influence cancer cell metabolism in vivo. Real-time flux analysis has been used to characterise metabolic vulnerabilities in a range of cancer cells, which have been successfully targeted in vivo [14, 15]. However, the methods used to identify metabolic vulnerabilities in cancer cells using real-time flux analysis have been ad hoc, and there are no clear stepwise protocols to identify metabolic vulnerabilities in cancer cells using this approach.

Breast cancer is highly diverse, with numerous different classifications based on immuno-profiles and the expression of specific growth factor receptors [16]. Different breast cancer classifications have a greater reliance on fatty acid [17] and glutamine [18] metabolism, suggesting that there is heterogeneity in metabolism between breast cancer subtypes. Although extensive genomic characterisation of different breast cancer types has been performed [19, 20], systematic assessment of the persistent metabolic alterations in breast cancer cells across its diverse classifications is limited. Therefore, the aim of the present study was to characterise the metabolic phenotypes across a panel of breast cancer cell lines before using a standardised, yet comprehensive, approach in an effort to identify potential metabolic vulnerabilities in major metabolic pathways coupled to ATP production using real-time metabolic flux analysis. These potential vulnerabilities were then targeted with specific metabolic inhibitors.

## Methods

### Cell culture

All human breast cancer cell lines and the MCF10a control cell line were obtained from American Type Culture Collection (ATCC), with the exception of the ESH172 line, which was a generous gift from Prof. Robin Anderson (Translational Breast Cancer Program, Olivia Newton-John Cancer Research Institute). All cell lines were cultured in growth media consisting of DMEM (4.5 g/l glucose; Invitrogen) supplemented with 10% foetal bovine serum (In Vitro Technologies) at 37 °C in 5% CO_2_. Cells were maintained at sub-confluence.

### Metabolic flux analysis

**Fig. 1:**
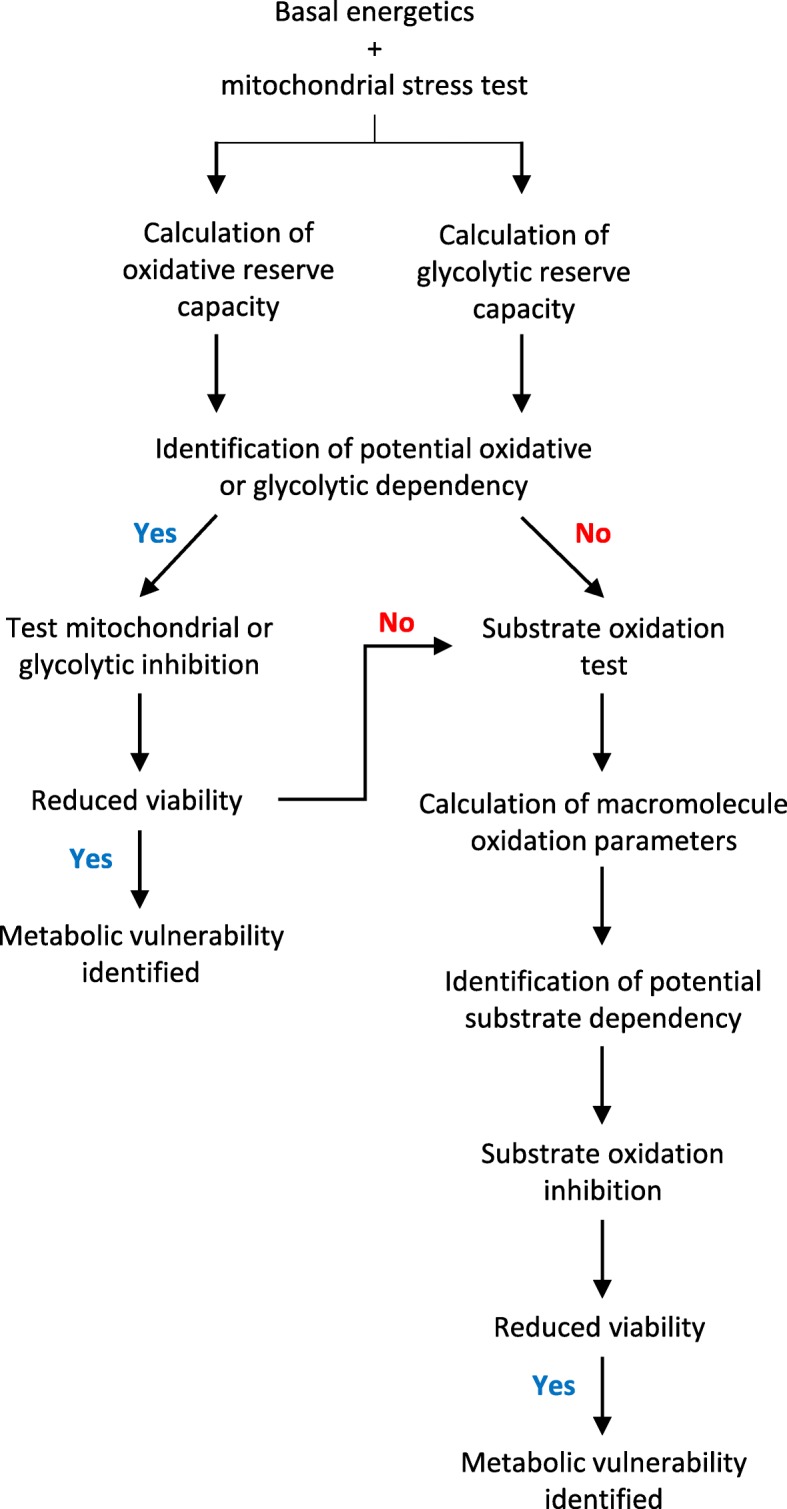
Systematic flux analysis protocol to identify targetable metabolic vulnerabilities in human breast cancer cell lines

A systematic flux approach to identify potential metabolic vulnerabilities in breast cancer cells was devised, which included assessment of basal bioenergetics, mitochondrial function and substrate oxidation dependency in a step-wise manner (Fig. 1).

Mitochondrial function was measured using the Seahorse XF24 Flux Analyser (Seahorse Bioscience), as we have previously described [21]. Briefly, the day prior to analysis, cells were seeded into a 24-well XF24 cell culture microplate (Seahorse Bioscience) such that they were ~ 80% confluent on the day of assay. Cells were washed and incubated in 600 μl assay running media (unbuffered DMEM, Invitrogen; supplemented with 25 mM glucose, 1 mM pyruvate and 1 mM glutamate, pH 7.4) in a non-CO_2_ incubator at 37 °C for 1 h before commencing the assay. Mitochondrial function was analysed by performing three baseline oxygen consumption rate (OCR) measurements, before a subsequent three measurements following injections of oligomycin (ATP synthase inhibitor; 1 μM final concentration), carbonyl cyanide-p-trifluoromethoxyphenylhydrazone (FCCP; mitochondrial oxidative phosphorylation uncoupler; 1 μM final concentration), rotenone (mitochondrial complex I inhibitor; 1 μM final concentration) and Antimycin A (mitochondrial complex III inhibitor; 1 μM final concentration). Each measurement cycle consisted of the following: 3 min mix, 3 min wait and 3 min measure. Extracellular acidification rate (ECAR) was measured concurrently with OCR. Data was normalised to total protein, which was determined after the assay using the bicinchoninic acid (BCA) method. Raw OCR and ECAR data plots are shown in Additional file 1: Figure S1.

**Fig. 2:**
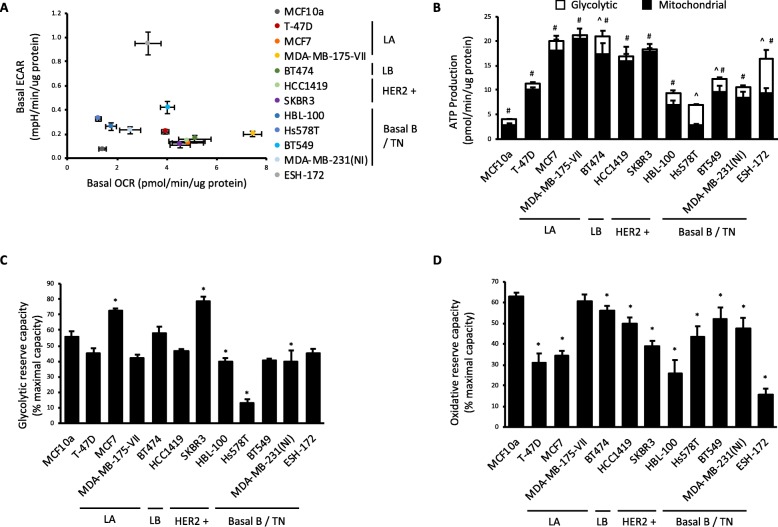
Human breast cancer cell lines are heterogeneous in their metabolic profiles. **a** Oxygen consumption rate (OCR) vs. extracellular acidification rate (ECAR). **b** Glycolytic and mitochondrial ATP production rate. **c** Glycolytic reserve capacity. **d** Oxidative reserve capacity. All data are mean ± SEM, *n* = 5–27 biological replicates/group. *p < 0.05 vs. MCF10a control cell line. Luminal A (LA), luminal B (LB), triple-negative (TN)

Basal OCR and basal ECAR (Fig. 2a) are the mean values of the three baseline measures. Rates of glycolytic and oxidative ATP production (Fig. 2b) were calculated using mean values from the three measurements of the relevant measurement cycle generated in this mitochondrial function assay as previously described [22]. Total glycolytic capacity was calculated as the mean value of three ECAR measurements following injection of oligomycin. Glycolytic reserve capacity (Fig. 2c) was subsequently calculated as percent difference between total glycolytic capacity and basal ECAR. Total oxidative capacity was calculated by subtracting the mean value of three measurements following Antimycin A injection, from the mean of the three measures following FCCP injection. Basal OCR attributed specifically to ATP production (i.e. excluding the contribution of H+ leak) was calculated by subtracting the mean value of three measurements following oligomycin injection from baseline OCR. Finally, oxidative reserve capacity (Fig. 2d) was calculated as the percent difference between total oxidative capacity and basal OCR.

### Testing metabolic vulnerabilities by inhibiting specific pathways

**Fig. 3:**
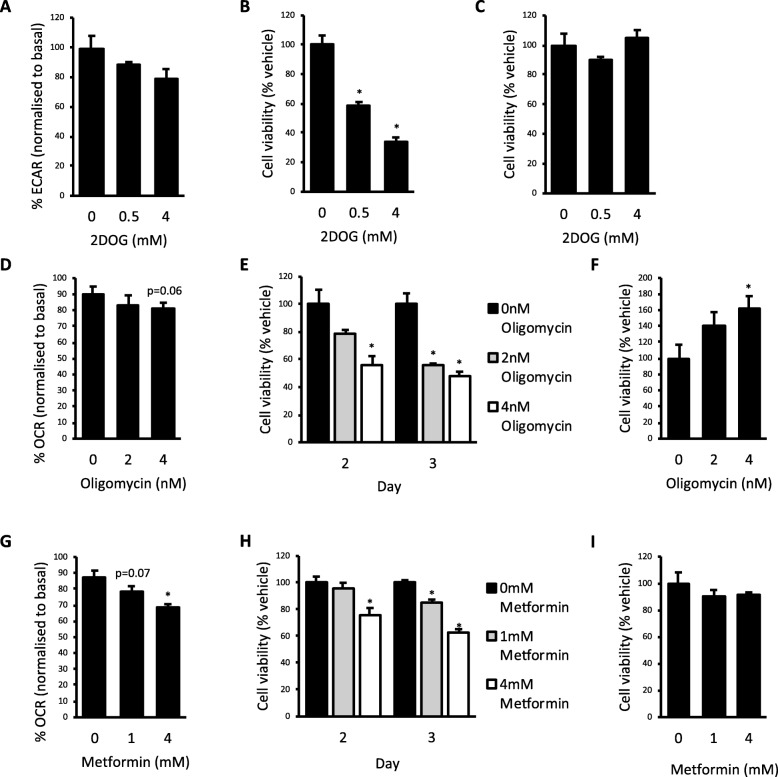
Targeting metabolic vulnerabilities reduced breast cancer cell viability. **a** Extracellular acidification rate (ECAR) in Hs578T cells treated acutely with 0.5 and 4 mM 2-deoxyglucose (2DOG). **b** Cell viability in Hs578T cells. **c** MCF10a cells treated with 0.5 and 4 mM 2DOG for 2 days. **d** Oxygen consumption rate (OCR) in ESH-172 cells treated acutely with 2 and 4 nM oligomycin. **e** Cell viability in ESH-172 cells treated with 2 and 4 nM oligomycin for 2 and 3 days. **f** Cell viability in MCF10a cells treated with 2 and 4 nM oligomycin for 3 days. **g** OCR in ESH-172 cells treated acutely with 1 and 4 mM metformin. **h** Cell viability in ESH-172 cells treated with 1 and 4 mM metformin for 2 and 3 days. **i** Cell viability in MCF10a cells treated with 1 and 4 mM metformin for 3 days. All data are mean ± SEM, *n* = 3–7 biological replicates/group. *p < 0.05 vs. vehicle

The effect of the metabolic inhibitors 2-deoxyglucose (2DOG; Fig. 3a), oligomycin (Fig. 3d) and metformin (Fig. 3g) on OCR or ECAR was assessed using the Seahorse XF24 Flux Analyser. The day before analysis, cells were seeded into a 24-well XF24 cell culture microplate (Seahorse Bioscience) such that they were ~ 80% confluent the following day. Cells were washed and incubated in 600 μl assay running media, as described above, prior to analysis. Three baseline OCR and ECAR measurements were obtained. The inhibitor of interest was then injected followed by eight more OCR and ECAR measurements. Each measurement cycle consisted of the following: 3 min mix, 2 min wait and 3 min measure. Data were normalised by dividing the final measurement following inhibitor injection, to ensure that the effects of subtle metabolic inhibition had plateaued, by the third baseline measure immediately prior to inhibitor injection. Raw data plots are shown in Additional file 1: Figure S2.

### Substrate utilisation analysis

The ability of the mitochondria to oxidise the macromolecules glucose, glutamine and palmitate was analysed using the Seahorse XF24 Flux Analyser (Fig. 5a–d) and the Mito Fuel Flex Test by Agilent, with some changes. Parameters measured by this assay are as follows: dependency—defined as the absolute reliance on the oxidation of a particular substrate for ATP production; capacity—defined as the maximal oxidation rate of a particular substrate; flexibility—defined as the ability to compensate mitochondrial oxidation by switching from one substrate to another; and residual oxidative capacity—defined as the maximal mitochondrial oxidation that can be achieved when the oxidation of one particular substrate has been inhibited.

To carry out the assay the day prior to analysis, cells were seeded into a 24-well XF24 cell culture microplate such that they were ~ 80% confluent the following day. Cells were washed and incubated in 600 μl assay running media (unbuffered DMEM, Invitrogen; supplemented with 5 mM glucose, 1 mM pyruvate, 1 mM glutamate and 0.5 mM carnitine, pH 7.4) at 37 °C in a non-CO_2_ incubator for 1 h prior to analysis. To measure dependency, three baseline OCR measurements were performed followed by five measurements after the injection of an inhibitor that targeted the pathway of interest (Table 1). A further five measurements were performed following the injection of inhibitors targeting the two alternative substrate oxidation pathways (Table 1). Dependency was calculated by subtracting the mean values of the OCR measurements taken following the first injection from the mean values of the basal OCR measurements. To measure capacity, three baseline OCR measurements were performed followed by five measurements after injection of the inhibitors targeting the two alternative substrate oxidation pathways, and a further five measurements after the injection of an inhibitor that targeted the pathway of interest. Capacity was calculated by subtracting the mean values of the OCR measurements following the second injection from those following the first injection. Flexibility was calculated by subtracting the dependency measurement from the capacity measurement for any given substrate. For the purpose of identifying potential metabolic vulnerabilities, we chose to also calculate the residual oxidative capacity. This was calculated by subtracting dependency from total oxidative capacity where total oxidative capacity is the mean values of the basal OCR measures subtracted by the mean values of the OCR measures following inhibition of all the oxidation pathways. This allowed the identification of substrates that cells were highly dependent upon, with little ability to use alternative pathways to compensate. Each measurement cycle consisted of the following: 3 min mix, 3 min wait and 3 min measure. Final concentrations of the inhibitors are as follows: 2 μM UK5099, 40 μM etomoxir and 3 μM Bis-2-(5-phenylacetamido-1,3,4-thiadiazol-2-yl)ethyl sulphide (BPTES). Raw data plots are shown in Additional file 1: Figure S3.

**Table 1:** Injection strategy of inhibitors for assessment of substrate oxidation dependency and capacity

Pathway	Measure	1st injection	2nd injection
Glucose	Dependency	UK5099	BPTES/etomoxir
	Capacity	BPTES/etomoxir	UK5099
Glutamine	Dependency	BPTES	Etomoxir/UK5099
	Capacity	Etomoxir/UK5099	BPTES
Palmitate	Dependency	Etomoxir	BPTES/UK5099
	Capacity	BPTES/UK5099	Etomoxir

### Cell viability assay

Crystal violet stain was used to quantify relative cell viability. Cells were seeded at sub-confluence into 96-well cell culture plates and treated with metabolic inhibitors the same day, once cells had adhered. Cells were allowed to proliferate for 2 to 3 days. Cells were then washed in PBS and then stained for 10 min at room temperature with 0.5% crystal violet (Sigma) in 30% ethanol. Wells containing no cells were included as a background control. Following staining, cells were washed three times with PBS before being lysed in 1% SDS. The crystal violet dye was dispersed by pipetting up and down, and absorbance was measured at a wavelength of 595 nm on an xMark microplate absorbance spectrophotometer (Bio-Rad Laboratories).

### Western blot analysis

For signalling analyses, cells were seeded into 12-well cell culture plates and treated with metabolic inhibitors the following day. After 2 days of treatment, protein was extracted using protein lysis buffer containing 50 mM Tris pH 7.5, 1 mM EDTA, 1 mM EGTA, 10% glycerol, 1% Triton X-100, 50 mM NaF, 5 mM Na4P2O7, 1 mM Na3VO4, 1 mM DTT and a protease inhibitor cocktail. Protein concentration was determined using a BCA Protein Assay kit (Pierce), and equal amounts of total protein were separated by SDS-PAGE. Proteins were transferred onto PVDF membrane and blocked for 1 h at room temperature with 1% BSA in Tris-buffered saline containing 0.05% Tween 20 (TBST, pH 7.4). Membranes were then incubated in the following primary antibodies overnight at 4 °C: phospho-AMPKα (Thr172) (Cell Signalling Technology), AMPKα (Cell Signalling Technology), phospho-mTOR (Ser2448) (Cell Signalling Technology), mTOR (Cell Signalling Technology), phospho-p70 S6 Kinase (Thr389) (Cell Signalling Technology), p70 S6 kinase (Cell Signalling Technology) and α-tubulin (Sigma-Aldrich). Membranes were then washed in TBST before being incubated for 1 h at room temperature with relevant HRP-conjugated secondary antibody used at 1:10,000 in TBST. The protein of interest was detected and visualised using Clarity Western ECL Substrate (Bio-Rad Laboratories) on a Chemidoc XRS System and Image Lab software (Bio-Rad Laboratories).

### Statistical analysis

Statistical analyses were performed using Prism GraphPad. Two-tailed *t* test or one-way ANOVA were used to compare groups as appropriate. Results are presented as mean ± SEM, and *p* < 0.05 was considered statistically significant.

## Results

### Identification of potential metabolic vulnerabilities in human breast cancer cell lines using glycolytic and oxidative flux measures

A panel of human breast cancer cell lines was assessed to first determine their basal metabolic profiles. Cell lines representing various immuno-profiles and classifications of the major breast cancer subtypes were analysed and compared to the control non-transformed breast epithelial MCF10a cell line. The classification of the ESH-172 cell line has not been extensively characterised [23]. Basal glycolytic (ECAR) and oxidative (OCR) flux was measured simultaneously in each cell line using the Seahorse XF24 Flux Analyser (Fig. 2a). This analysis revealed a high level of heterogeneity between cell lines in both measures. Compared with MCF10a cells, all breast cancer cell lines had elevated basal energetics, represented by increased glycolysis and oxidative cellular respiration. Using data generated in subsequent mitochondrial function tests, the rate of ATP production from glycolytic and oxidative sources was also calculated. All breast cancer cell lines produced greater amounts of ATP than MCF10a cells through oxidative pathways, with the exception of the Hs578T line (Fig. 2b). In contrast, only the BT474, Hs578T, BT549 and ESH-172 cell lines produced more ATP than MCF10a cells through glycolysis (Fig. 2b). Additional analyses were performed to identify cell lines with limited reserve capacity in either glycolytic (Fig. 2c) or oxidative flux (Fig. 2d) in the basal state. We reasoned that any cell line using a high proportion of its total flux capacity for a particular pathway could represent a potential metabolic vulnerability. Although most cell lines possessed between 40 and 60% glycolytic reserve capacity, the Hs578T cell line was using in excess of 90% of its total glycolytic capacity, leaving only ~ 10% in reserve capacity (Fig. 2c). Similarly, assessment of oxidative reserve capacity revealed that the ESH-172 cell line possessed only ~ 10% reserve capacity, the lowest of all cell lines analysed (Fig. 2d).

### Targeting metabolic vulnerabilities to reduce cell viability

As the Hs578T and ESH-172 cell lines used glycolysis and oxidative metabolism, respectively, at close to maximal flux capacity in the basal state, we next examined whether these could be a druggable vulnerability in these cells. By identifying metabolic pathways with little reserve flux capacity, we reasoned that even minor inhibition of these pathways could have discernible effects on cell viability. To assess whether inhibition of the glycolytic pathway in Hs578T cells is a metabolic vulnerability, cells were treated with 2DOG, which provides feedback inhibition to the hexokinase/glucokinase reaction and slows glycolytic flux [24]. Acute treatment with 0.5 mM and 4 mM 2DOG resulted in a dose-dependent decrease in ECAR; however, this effect was not statistically significant (Fig. 3a). Following 2 days of 0.5 mM and 4 mM 2DOG treatment, there was a dose-dependent decrease in Hs578T cell viability by 41% and 66%, respectively, compared to vehicle control (Fig. 3b). To ensure this was a cell line-specific effect, MCF10a cells were also treated with 2DOG for 2 days and there was no significant effect on viability (Fig. 3c), suggesting that mild glycolytic inhibition is not a metabolic vulnerability in these cells.

We next sought to determine whether mild inhibition of oxidative ATP generation impacts the viability of ESH-172 cells. When these cells were acutely treated with 2 or 4 nM of the ATP synthase inhibitor oligomycin, a small but non-statistically significant reduction in OCR was observed (Fig. 3d). Viability was significantly reduced by 44% at day 2 of treatment with 4 nM oligomycin, and 44% and 52% at day 3 of treatment with 2 nM and 4 nM oligomycin, respectively (Fig. 3e). Interestingly, treatment of control MCF10a cells with 4 nM oligomycin for 3 days increased cell viability (Fig. 3f). These data show that mild inhibition of oxidative ATP generation with oligomycin reduced cell viability specifically in ESH-172 cells. As irreversible mitochondrial inhibitors such as oligomycin cannot be used clinically, we next assessed whether treatment of ESH-172 cells with metformin had similar effects on viability. Metformin is the most widely prescribed anti-diabetic agent and an inhibitor of complex I in the electron transport chain that reduces oxidative ATP generation [25]. Furthermore, a number of studies have found that metformin administration reduces breast cancer risk [26, 27]. ESH-172 cells were treated acutely with 1 mM and 4 mM metformin, and OCR was significantly reduced with 4 mM treatment (Fig. 3g). ESH-172 viability was decreased by 24% at day 2 of treatment with 4 mM metformin and by 15% and 37% at day 3 of treatment with 1 mM and 4 mM metformin, respectively (Fig. 3h). Metformin treatment had no effect on the viability of MCF10a cells after 3 days of treatment (Fig. 3i). These data suggest that metformin reduced cell viability specifically in ESH-172 breast cancer cells.

### Effect of metabolic inhibitors on AMPK and mTORC1 signalling

**Fig. 4:**
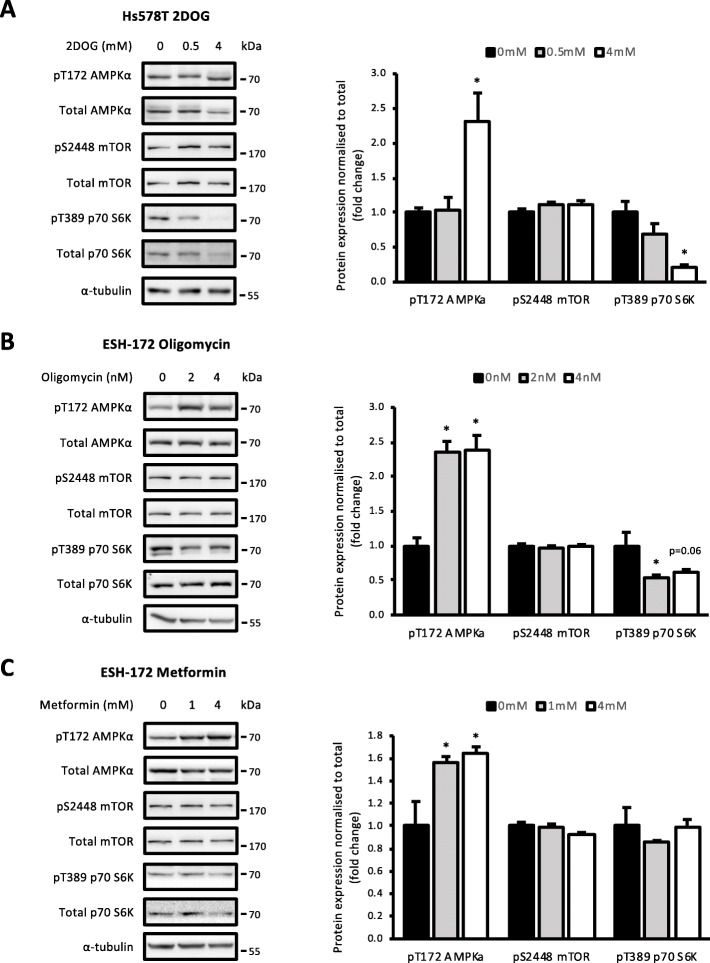
Cellular energy homeostasis is disrupted with metabolic inhibitors. AMPK-mTORC1 signalling in **a** Hs578T cells treated with 0.5 and 4 mM 2DOG for 2 days, **b** ESH-172 cells treated with 2 and 4 nM oligomycin for 2 days and **c** ESH-72 cells treated with 1 and 4 mM metformin for 2 days. All data are mean ± SEM, *n* = 3 biological replicates/group. *p < 0.05 vs. vehicle

The metabolic vulnerabilities in the Hs578T and ESH-172 cells were identified due to their high contribution to ATP production in those cell lines. Therefore, it was predicted that targeting these metabolic vulnerabilities would induce an energetic stress that impacts on cancer cell growth signalling. This could lead to AMPK activation, which is known to inhibit mTORC1 signalling, including the mTORC1 substrate p70 S6K, through multiple mechanisms [28]. The effect of targeting these metabolic vulnerabilities on this signalling axis was explored. Hs578T cells treated with 4 mM 2DOG for 2 days increased pT172 AMPKα compared with vehicle and decreased pT389 p70 S6K, indicating a deficient ATP supply that impacts on growth signalling (Fig. 4a). There was no effect on phosphorylation of mTOR at S2448 with either dose (Fig. 4b). Treatment of ESH-172 cells with oligomycin for 2 days significantly increased pT172 AMPKα at both 2 and 4 nM doses and decreased pT389 p70 S6K at both doses; however, this change was not significant (*p* = 0.079 and 0.125, respectively; Fig. 4b). Again, pS2448 mTOR showed no change compared to vehicle (Fig. 4b). Metformin treatment of ESH-172 cells increased AMPKα T172 phosphorylation at 4 mM after 2 days of treatment (Fig. 4c). Both pS2448 mTOR and pT389 p70 S6K were not affected at either dose of metformin (Fig. 4c). These data show that targeting metabolic vulnerabilities with metabolic inhibitors induced AMPK activation and impaired mTORC1 signalling, while metformin activated AMPK but did not affect mTORC1 activity.

### Macromolecule substrate oxidation dependency as potential metabolic vulnerabilities

**Fig. 5:**
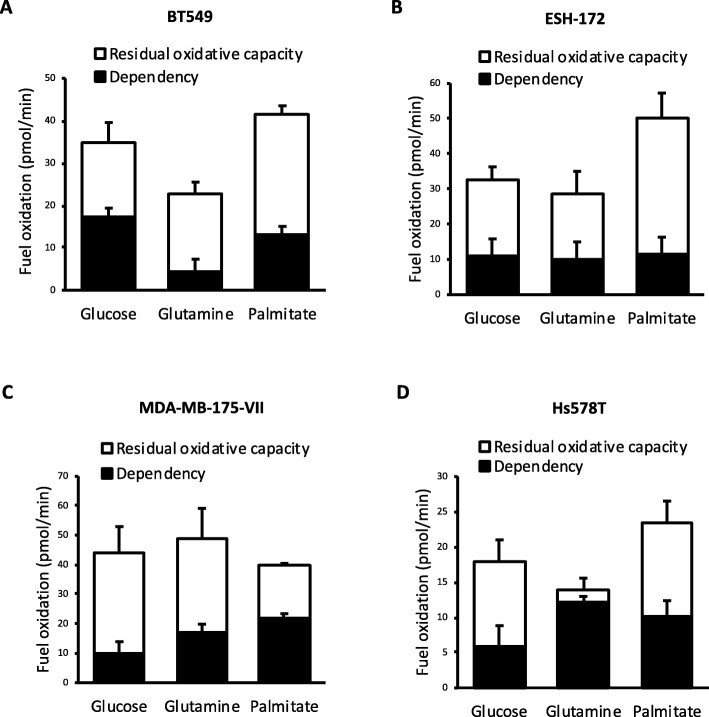
Hs578T cells are heavily reliant on glutamine oxidation. The dependency of mitochondria on the oxidation of glucose, glutamine or palmitate and residual oxidative capacity of alternate substrates in **a** BT549, **b** ESH-172, **c** MDA-MB-175-VII and **d** Hs578T cells. All data are mean ± SEM, *n* = 3–5 biological replicates/group

The flux approach taken to date identified potential metabolic vulnerabilities based on the overreliance on glycolytic or oxidative metabolism to produce ATP. As these measures alone did not detect obvious potential vulnerabilities in all cell lines, we next examined whether potential vulnerabilities could be identified through the overreliance on the oxidation of any one of the major macromolecules. To test this concept, cell lines were selected based on their oxidative and glycolytic profiles. The BT549 cell line was selected as a mid-range oxidative and glycolytic line, ESH-172 cells as a highly glycolytic line, MDA-MB-175-VII cells as a highly oxidative line and Hs578T cells as a low-range oxidative line. The dependency of cell lines on glucose, glutamine and palmitate oxidation to drive mitochondrial respiration was examined, and a potential vulnerability was identified where a cell line had limited residual capacity to oxidise the two alternative macromolecules. The BT549 (Fig. 5a), ESH-172 (Fig. 5b) and MDA-MB-175-VII cell lines (Fig. 5c) were not dependent on the oxidation of any one macromolecule. In contrast, the Hs578T cell line was found to be highly dependent on glutamine oxidation, with limited residual capacity to oxidise palmitate and/or glucose (Fig. 5c), which could be a targetable vulnerability.

### Inhibition of glutamine oxidation to reduce cell viability

**Fig. 6:**
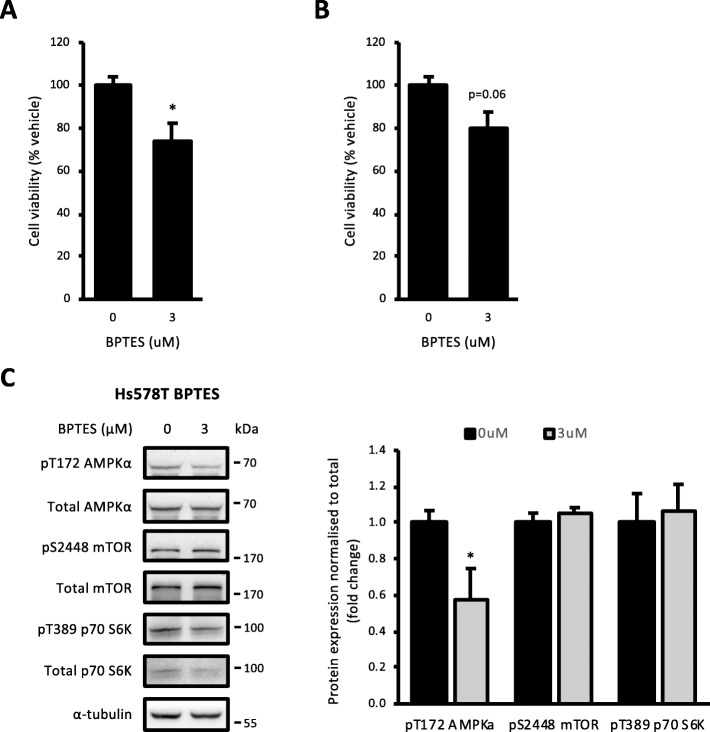
Inhibition of glutamine oxidation reduced Hs578T cell viability. **a** Cell viability in Hs578T cells treated with 3 μM BPTES for 2 days. **b** Cell viability in MCF10a cells treated with 3 μM BPTES for 2 days. **c** AMPK-mTORC1 signalling in Hs578T cells treated with 3 μM BPTES for 2 days. All data are mean ± SEM, *n* = 3–4 biological replicates/group. *p < 0.05 vs. vehicle

To assess the dependency of the Hs578T cell line on glutamine metabolism and its potential as a metabolic target, we treated these cells with BPTES. This compound is an inhibitor of the glutaminase enzyme, which is responsible for the conversion of glutamine to glutamate following glutamine uptake [29]. Treatment of Hs578T cells with 3 μM BPTES for 2 days reduced viability by 25% relative to vehicle control (Fig. 6a). The same treatment had similar trends towards reducing the viability of MCF10a cells (Fig. 6b). Although this was not statistically significant, it likely reflects the key role for glutamine metabolism in most proliferating cell types. The inhibition of glutamine metabolism on growth signalling was also investigated. Hs578T cells treated with 3 μM BPTES for 2 days had decreased pT172 AMPKα expression compared to vehicle, but there was no change in pS2448 mTOR or pT389 p70 S6K expression compared to vehicle (Fig. 6c). These data suggest that inhibition of glutamine metabolism impairs viability in Hs578T cells through alternate mechanisms.

## Discussion

Breast tumours are extensively heterogeneous in their growth, metastatic potential and metabolism, even within classifications. As our understanding of this heterogeneity increases so too does the realisation that individualised treatments may be necessary for improved patient outcomes. Hence, the ability to culture breast tumour cells ex vivo in order to identify vulnerabilities that can be exploited could prove to be a powerful tool in cancer treatment [30]. In the present study, we analysed the metabolic profiles of a panel of breast cancer cell lines that spanned the different breast cancer classifications and molecular subtypes using real-time metabolic flux analysis. Despite high metabolic heterogeneity, this analysis allowed us to identify targetable metabolic vulnerabilities in major metabolic pathways, specifically linked to ATP production, in order to reduce the relative viability of a number of different breast cancer cell lines. Although we did not determine whether these effects were due to inhibition of cell proliferation, induction of cell death or both, this approach paves the way for more mechanistic studies examining these interactions.

In the present study, basal glycolytic and oxidative metabolic flux analysis of the various breast cancer cell lines revealed that their energetic profile is vastly heterogeneous. Compared with MCF10a control cells, the majority of breast cancer cell lines had increased oxidative respiration rate, while just four cell lines had elevated rates of glycolytic ATP production. Increased glycolytic rate has long been established as an adaptive response of cancer cells, regardless of oxygen availability [2]. This phenomenon, known as the ‘Warburg effect’, provides not only ATP, but also metabolic intermediates from biosynthetic pathways that stem from the glycolytic pathway to support rapid proliferation and survival of the cancer cells [1]. An interesting finding from the present study was that the glycolytic pathway produces very little ATP in most breast cancer cell types, suggesting that glucose catabolism through this pathway is more closely linked to biosynthetic processes. Increased oxidative respiration also supports proliferation and survival by serving as the major source of ATP for the cell [31–33], and our analyses showed that most breast cancer cells relied predominantly on oxidative metabolism for their ATP needs under normoxic conditions. Moreover, breast cancer subtypes generally had no discernible common metabolic profile based on these measures. However, it should be noted that three of the four cell lines with elevated rates of glycolytic ATP production were basal B/triple negative cell lines. Glycolytic inhibition has previously been used to reduce viability of breast cancer cells from this classification [34], both under normoxic and hypoxic conditions [35]. These studies support the approach taken in the present study, and although culture conditions could be optimised to replicate in vivo conditions, flux approaches to identify metabolic vulnerabilities appear to have efficacy under standard culturing conditions.

Given the heterogeneity of the basal energetic profile of the cell lines studied here, individualised examination of cellular metabolic measures in vitro may be necessary to identify potential vulnerabilities that could be exploited to reduce proliferation and/or survival of these cells. Indeed, flux profiling of patient-derived cancer cells could allow personalised treatment. In order for this to be a viable approach, it will be critical to understand whether persistent metabolic reprogramming events are retained in patient cell lines ex vivo. Notwithstanding, the systematic flux analysis used here was able to identify cell lines that were heavily reliant on ATP generation through either glycolysis or oxidative respiration, and targeting these respective pathways in predicted vulnerable cell lines reduced their viability by inducing an energetic crisis, without effect on control cells. We found that treatment of these breast cancer cell lines with metabolic inhibitors to reduce flux through either glycolysis or oxidative phosphorylation resulted in the activation of AMPK and inhibition of mTORC1 signalling. AMPK is a cellular energy sensor that monitors ATP/AMP and ATP/ADP ratios and is activated through phosphorylation to increase ATP production and meet the energetic demands of the cell [28, 36]. Downstream inactivation of p70 S6K was also observed in these cells where p70 S6K plays a role in protein synthesis and cell growth [37, 38]. Furthermore, the energetic imbalance observed in these cells following metabolic inhibition as assessed by AMPK activation suggests that they were not able to upregulate other pathways to compensate for the reduction in ATP levels. Indeed, when these measures were analysed, cells treated with various metabolic inhibitors did not increase flux through the alternative major ATP-producing pathway. This is of interest as metabolic adaption to unfavourable environments is a hallmark of cancer cells and often metabolic inhibition cannot be used as a monotherapy but rather to sensitise cells to a further insult [39].

Although the ATP synthase inhibitor oligomycin was effective at reducing viability of ESH-172 cells, mitochondrial inhibitors such as this cannot be used clinically due to their toxicity. An alternative therapy that is reasonably well tolerated in humans is the anti-diabetic drug metformin, which can act as an inhibitor of complex I at high concentrations that reduces oxidative ATP generation [25]. Metformin reduced ESH-172 cell viability, which is consistent with evidence that metformin exerts anti-cancer effects in breast tumours [26, 27, 40–42]. However, in a clinical setting, the response to metformin varies widely between individuals and is often used as a combination therapy [43]. We found that in ESH-172 cells, metformin treatment significantly reduced the viability of the cells relative to vehicle control without effects on the viability of control MCF10a cells. Although the mechanism of action of metformin is yet to be clearly defined, it is accepted that it acts as an inhibitor of complex I in the ETC [25, 44, 45] and can therefore reduce ATP turnover resulting in the activation of AMPK [46]. Our findings are consistent with this as AMPK was activated with metformin treatment; however, there was no concomitant reduction in p70 S6K activation suggesting an alternative downstream mechanism independent of mTORC1 inhibition. Although this finding is in contrast to the current literature suggesting that metformin can reduce protein synthesis and proliferation through the inhibition of mTOR and p70 S6K [47–49], it is consistent with results from an in vitro study by Hadad et al. [50]. This study found that despite increased activation of AMPK in response to metformin, phosphorylation of p70 S6K in the human breast cancer cell lines MCF-7 (ER-positive) and MDA-MB-231 (ER-negative) was unchanged [50]. Instead, increased phosphorylation of acetyl-CoA carboxylase (ACC) by AMPK was identified as an alternative mechanism following metformin treatment potentially leading to reduced lipid synthesis [50], which may also be relevant in the present study. Further highlighting the complexity in the cellular metformin response, Queiroz et al. showed that metformin increased mitochondrial ROS production and activated FOXO3a in MCF7 cells, which was associated with an increase in p27 and cell cycle arrest [49]. Metformin has also been found to challenge the viability of various cancer cells through regulation of p53 activity, Wnt/β-catenin signalling [51] and mitochondrial mediators of apoptosis [52]. This suggests that the cellular responses to metformin appear to be cell type and context dependent and additional research will be required to establish the mechanism of action in ESH-172 cells.

The approach used in the present study could potentially identify additional cell lines with similar metabolic vulnerabilities beyond those reported. For example, the HBL-100 cell line may also be sensitive to oxidative respiration inhibitors, as it too has little oxidative reserve capacity. However, it is reasonable to conclude that quantification of the reserve capacity of major ATP-producing pathways may not be effective at identifying vulnerabilities in all the cell lines. We therefore sought to broaden the measures available to identify potential metabolic vulnerabilities by examining the reliance of particular cell lines on the oxidation of the major macromolecules. The Hs578T cell line was identified as being highly dependent on glutamine oxidation with it accounting for a large amount of its total oxidative capacity. Importantly, this finding provides some validation for the approach, as glutamine utilisation has previously been identified as a metabolic vulnerability in triple-negative breast cancer cells [18]. In the present study, inhibition of glutamine oxidation in Hs578T cells, by treating with BPTES, decreased cell viability relative to vehicle control-treated cells and was associated with a decrease in AMPK phosphorylation and no effect on the activation of p70 S6K. As AMPK was not activated by BPTES treatment, this suggests that inhibition of glutamine oxidation does not induce an energetic crisis, but could potentially change other aspects of cellular bioenergetics [53], thus adversely effecting viability. Indeed, glutaminase inhibition also tended to negatively impact viability in control MCF10a cells. It should be noted that the concentrations of BPTES in these viability assays were the same used to completely suppress glutamine oxidation in substrate oxidation assays. Therefore, titration of BPTES might reveal a therapeutic concentration with efficacy in reducing cancer cell viability, but not that of non-transformed cells. An interesting observation from this approach was that the multiple measures of total oxidative capacity varied between the different substrate dependency tests. A technical limitation of this approach using the XF24 system is that each substrate needs to be considered in an independent assay. Whether the differences in total oxidative capacity between these assays are due to inter-assay variability or other biological factors, such as circadian regulation of metabolic flux, needs to be determined.

An important limitation of this study is that cells were not grown in conditions replicating those encountered in vivo, where changes in oxygen tension and nutrient availability induce changes in the metabolic profile of cancer cells. It must also be recognised that cells in culture may have undergone epigenetic changes over time [54] that could influence their bioenergetic profile. However, the methods described here to systematically identify metabolic vulnerabilities provide important proof-of-concept evidence for this approach. As a key feature of cancer cells is metabolic flexibility, the ability to identify a particular pathway or macromolecule that is heavily relied upon, and with little spare capacity, to fuel the cell has been shown here to be a targetable vulnerability. Further studies of tumour cells isolated from animal models will be necessary to determine whether this method could be used in patients.

## Conclusion

Breast cancer cells display heterogeneous metabolic profiles even within the same classification; however, systematic flux profiling can reveal targetable metabolic vulnerabilities in individual cell lines.

## Supplementary information

**Additional file 1: Figure S1.** (A) Oxygen consumption rate (OCR) and (B) extracellular acidification rate (ECAR) raw data plots of mitochondrial function assay in human breast cancer cell lines and the MCF10a cell line. All data are mean ± SEM, *n* = 10–37 biological replicates/group. **Figure S2.** (A) Extracellular acidification rate (ECAR) raw data plot of Hs578T cells treated acutely with 0.5 and 4 mM 2-deoxyglucose (2DOG). (B) Oxygen consumption rate (OCR) raw data plot of ESH-172 cells treated acutely with 2 and 4 nM oligomycin. (C) OCR raw data plot of ESH-172 cells treated acutely with 1 and 4 mM metformin. All data are mean ± SEM, *n* = 3–7 biological replicates/group. **Figure S3.** Oxygen consumption rate (OCR) raw data plots used to define dependency on glucose, glutamine and palmitate oxidation in (A) BT549; (B) ESH-172; (C) MDA-MB-175-VII, and; (D) Hs578T cells. Inhibitors used in first and second injections are described in Table 1. All data are mean ± SEM, *n* = 5 biological replicates/group.

## Data Availability

Data sharing is not applicable to this article as no datasets were generated or analysed during the current study.

## Abbreviations

2DOG: 2-Deoxyglucose
ACC: Acetyl-CoA carboxylase
BCA: Bicinchoninic acid
BPTES: Bis-2-(5-phenylacetamido-1,3,4-thiadiazol-2-yl)ethyl sulfide
ECAR: Extracellular acidification rate
ETC: Electron transport chain
FCCP: Carbonyl cyanide-p-trifluoromethoxyphenylhydrazone
mTORC1: Mammalian target of rapamycin complex 1
OCR: Oxygen consumption rate
TBST: Tris-buffered saline containing 0.05% Tween 20
