# Supplementary material for: A systematic flux analysis approach to identify metabolic vulnerabilities in human breast cancer cell lines
Source: Cancer Metab. 2019 Dec 27;7:12. doi: 10.1186/s40170-019-0207-x (PMC6935091; doi:10.1186/s40170-019-0207-x)
Supplement: Supplementary file 1 — Additional file 1: Figure S1. (A) Oxygen consumption rate (OCR) and (B) extracellular acidification rate (ECAR) raw data plots of mitochondrial function assay in human breast cancer cell lines and the MCF10a cell line. All data are mean ± SEM, n = 10–37 biological replicates/group. Figure S2. (A) Extracellular acidification rate (ECAR) raw data plot of Hs578T cells treated acutely with 0.5 and 4 mM 2-deoxyglucose (2DOG). (B) Oxygen consumption rate (OCR) raw data plot of ESH-172 cells treated acutely with 2 and 4 nM oligomycin. (C) OCR raw data plot of ESH-172 cells treated acutely with 1 and 4 mM metformin. All data are mean ± SEM, n = 3–7 biological replicates/group. Figure S3. Oxygen consumption rate (OCR) raw data plots used to define dependency on glucose, glutamine and palmitate oxidation in (A) BT549; (B) ESH-172; (C) MDA-MB-175-VII, and; (D) Hs578T cells. Inhibitors used in first and second injections are described in Table 1. All data are mean ± SEM, n = 5 biological replicates/group. [file 40170_2019_207_MOESM1_ESM.pdf]

Supplementary information for:

A systematic flux analysis approach to identify metabolic vulnerabilities in human breast cancer cell lines

Sheree D. Martin[1], Sean L. McGee[1]

Supplementary Figure 1

A

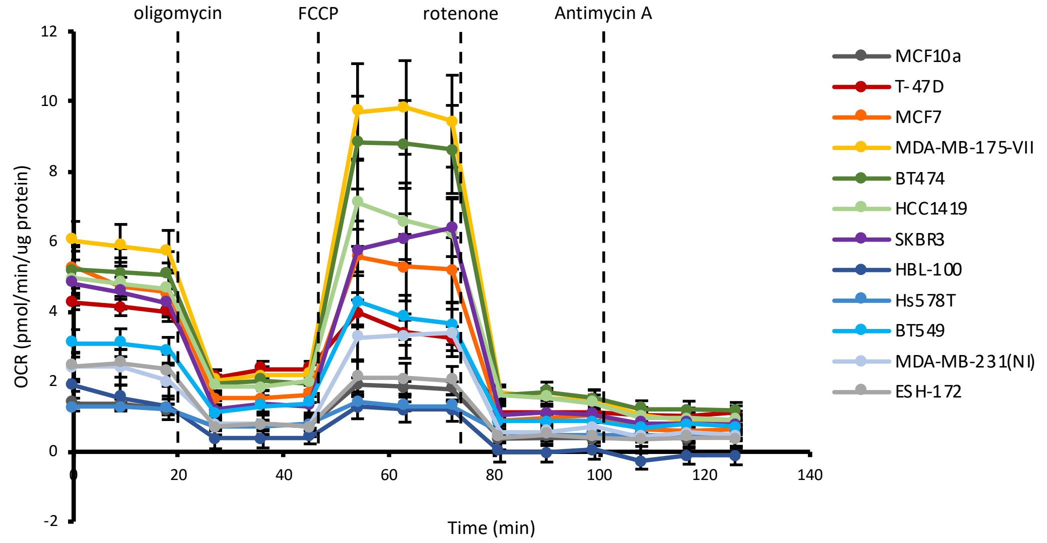

B

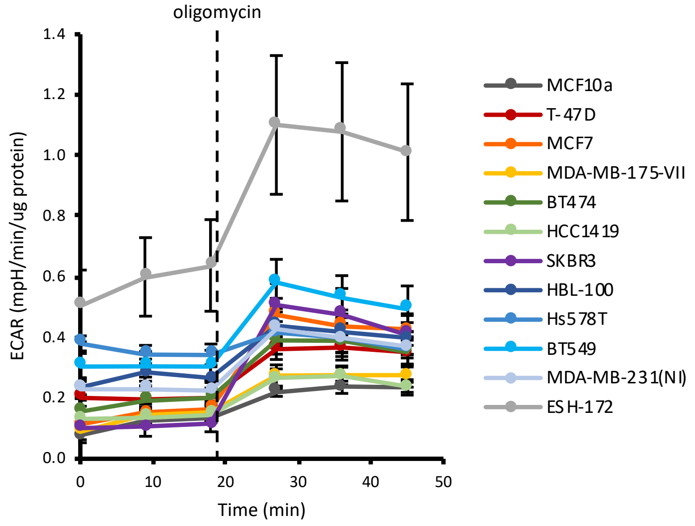

Supplementary Figure 1.

(A) Oxygen consumption rate (OCR) and (B) extracellular acidification rate (ECAR) raw data plots of mitochondrial function assay in human breast cancer cell lines and the MCF10a cell line. All data are mean ± SEM, n = 10-37 biological replicates/group.

Supplementary Figure 2

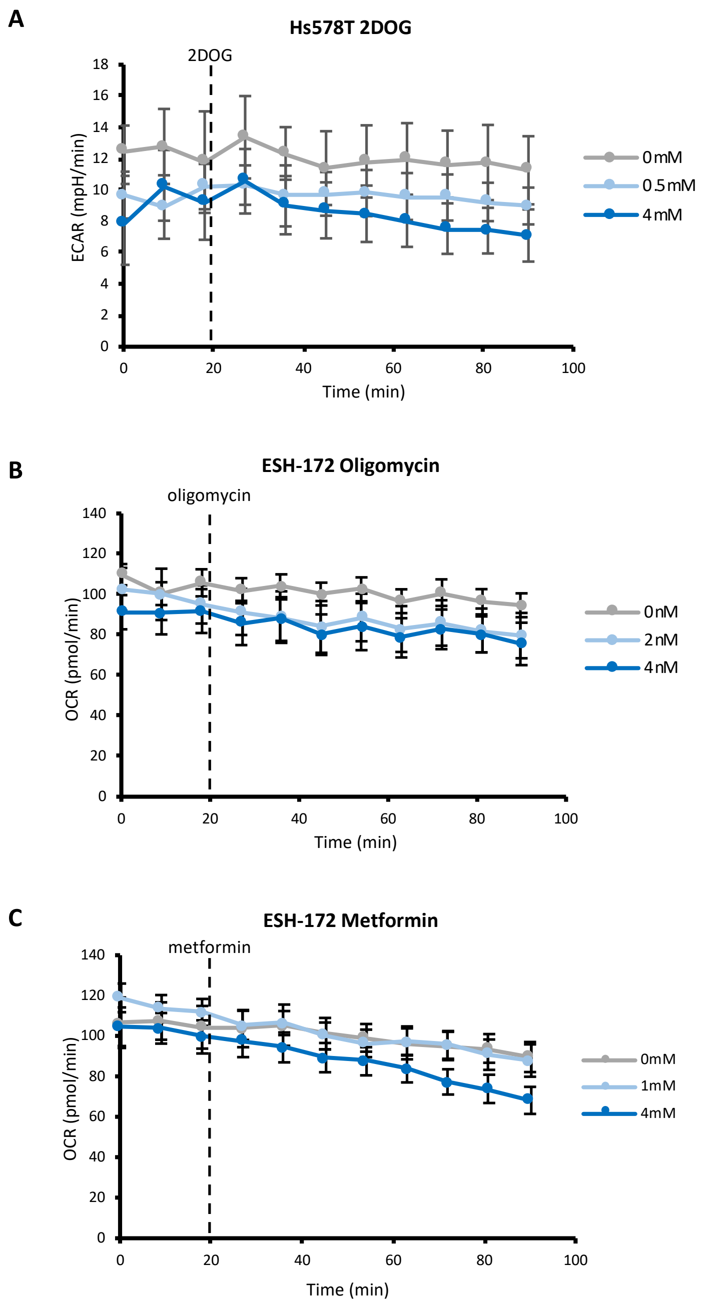

Supplementary Figure 2.

(A) Extracellular acidification rate (ECAR) raw data plot of Hs578T cells treated acutely with 0.5 and 4mM 2-deoxyglucose (2DOG). (B) Oxygen consumption rate (OCR) raw data plot of ESH-172 cells treated acutely with 2 and 4nM oligomycin. (C) OCR raw data plot of ESH-172 cells treated acutely with 1 and 4mM metformin. All data are mean ± SEM, n = 3-7 biological replicates/group.

Supplementary Figure 3

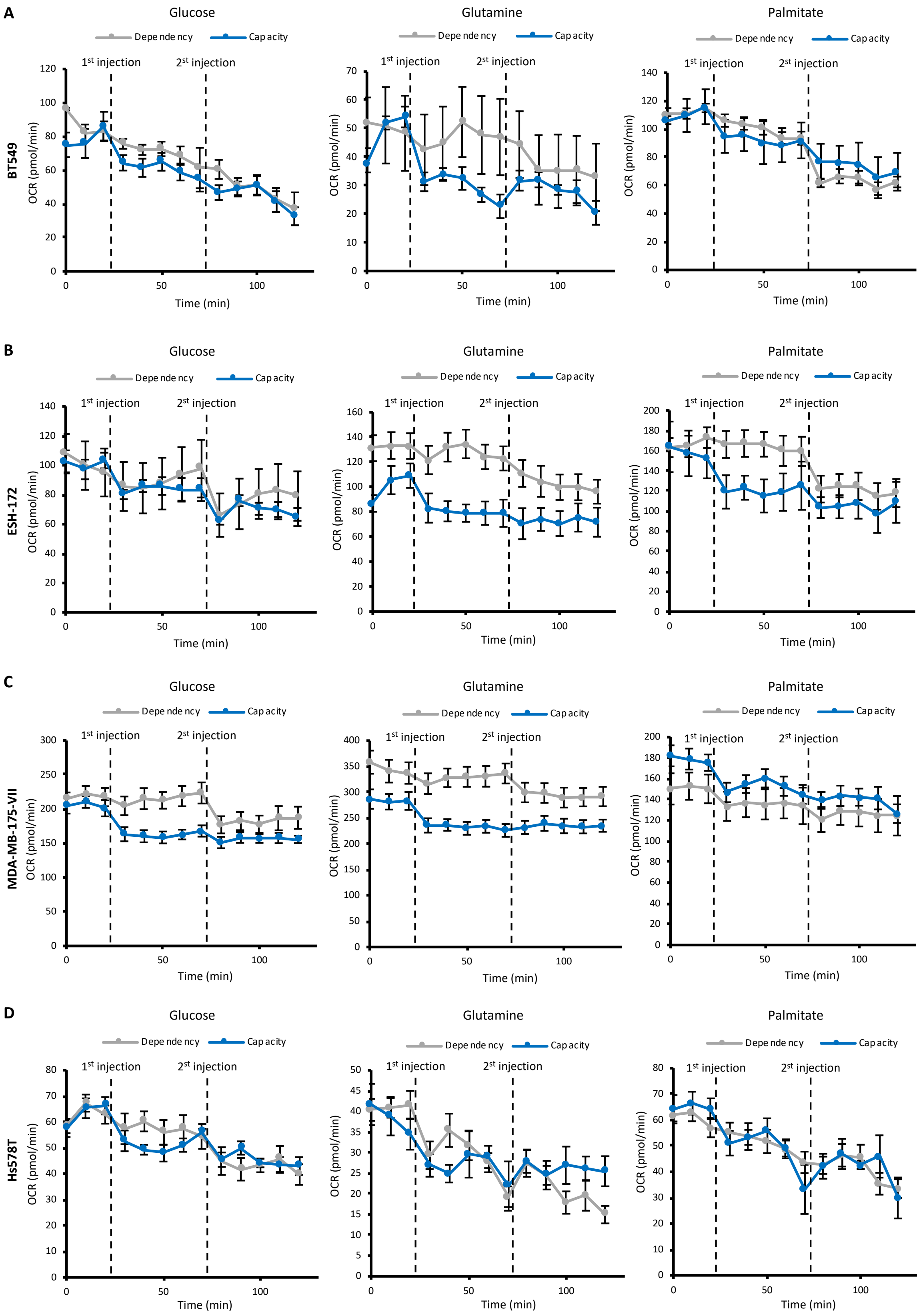

Supplementary Figure 3.

Oxygen consumption rate (OCR) raw data plots used to define dependency on glucose, glutamine and palmitate oxidation in (A) BT549; (B) ESH-172; (C) MDA-MB-175-VII, and; (D) Hs578T cells. Inhibitors used in first and second injections are described in Table 1. All data are mean ± SEM, n = 5 biological replicates/group.
